# Elucidating the 16S rRNA 3′ boundaries and defining optimal SD/aSD pairing in *Escherichia coli* and *Bacillus subtilis* using RNA-Seq data

**DOI:** 10.1038/s41598-017-17918-6

**Published:** 2017-12-15

**Authors:** Yulong Wei, Jordan R. Silke, Xuhua Xia

**Affiliations:** 1 Department of Biology, University of Ottawa, 30 Marie Curie, P.O. Box 450, Station A, Ottawa, Ontario Canada; 2 Ottawa Institute of Systems Biology, Ottawa, Ontario K1H 8M5 Canada

## Abstract

Bacterial translation initiation is influenced by base pairing between the Shine-Dalgarno (SD) sequence in the 5′ UTR of mRNA and the anti-SD (aSD) sequence at the free 3′ end of the 16S rRNA (3′ TAIL) due to: 1) the SD/aSD sequence binding location and 2) SD/aSD binding affinity. In order to understand what makes an SD/aSD interaction optimal, we must define: 1) terminus of the 3′ TAIL and 2) extent of the core aSD sequence within the 3′ TAIL. Our approach to characterize these components in *Escherichia coli* and *Bacillus subtilis* involves 1) mapping the 3′ boundary of the mature 16S rRNA using high-throughput RNA sequencing (RNA-Seq), and 2) identifying the segment within the 3′ TAIL that is strongly preferred in SD/aSD pairing. Using RNA-Seq data, we resolve previous discrepancies in the reported 3′ TAIL in *B. subtilis* and recovered the established 3′ TAIL in *E. coli*. Furthermore, we extend previous studies to suggest that both highly and lowly expressed genes favor SD sequences with intermediate binding affinity, but this trend is exclusive to SD sequences that complement the core aSD sequences defined herein.

## Introduction

Protein production is a highly controlled and optimized process in bacterial species^1^, and translation initiation is often recognized as the rate-limiting step of the translation process^2–4^. As such, finding ways to overcome this bottleneck in efficiency is important for using bacteria in transgenic biosynthesis of important pharmaceutical compounds such as insulin^5^. Translation initiation efficiency in bacteria is strongly influenced by the binding affinity between the Shine-Dalgarno (SD) sequence upstream of the start codon on mRNA and the anti-SD (aSD) sequence located at the free 3′ end of the 16S rRNA (3′ TAIL)^6,7^. Furthermore, the location of the SD/aSD interaction relative to the start codon must also be considered to ensure that the pairing positions the ribosomal P-site at the start codon^6–9^.

**Figure 1:**
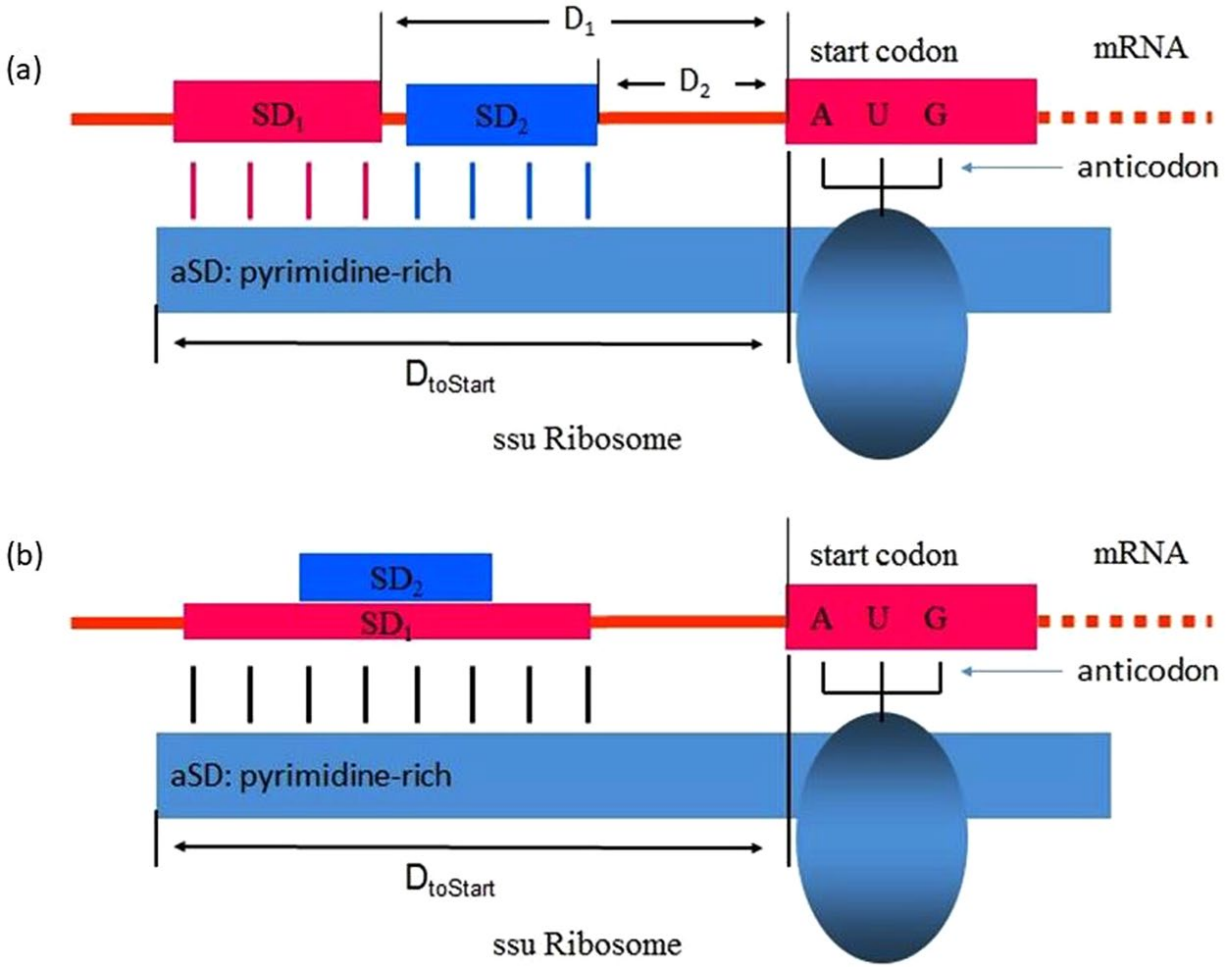
Schematic model of SD/aSD interaction, illustrating D_toStart_ (**a** and **b**), difference in the two “leash” distances (D1 and D2) and in binding affinity (**b**) between two SD/aSD interactions involving SD_1_ and SD_2_.

A recent model of SD/aSD interaction^10,11^ (Fig. 1) suggests that optimal SD/aSD pairing may depend on three factors: 1) D_toStart_ (Fig. 1) which specifies the distance, in nucleotides, between the 16S rRNA 3′ terminus and the start codon, 2) SD/aSD binding affinity (Figs 1b), and 3) “leash” distance measured by D_1_ and D_2_ (Fig. 1). D_toStart_ is strongly constrained within a narrow range. Intra-strand secondary structure that embeds the SD sequence is also known to affect SD/aSD function in localizing translation initiation codon^11,12^. Characterizing these features demands the precise terminus of the 16S rRNA which is often unclear, as is the case for *Bacillus subtilis*.

### RNA-seq data as a novel approach to define the 3′ TAIL in *E. coli* and *B. subtilis*

The 3′ TAIL was previously reported to be 5′-CCUCCUUUCU-3′^13^ based on personal communication between the authors and Carl Woese, although no explicit data to substantiate the terminus of the 3′ TAIL in *B*. *subtilis* was published. Acceptance of the 5′-CCUCCUUUCU-3′ end^13,14^ arose because Woese and colleagues published the details of their RNA sequencing method^15^ as well as the 3′ TAILs in a number of bacterial species^16^. Since then, alternative rDNA annotations of the *B. subtilis* 3′ TAIL have emerged, including 5′-CCUCCUUUCUA-3′ (NC_000964)^17^ and 5′-CCUCCUUUCUAA-3′ (NZ_CP010052) which have been used in recent studies on *B. subtilis* 16S rRNA^9,18^. Discrepancies in these reported 3′ TAILs likely arose due to the fact that multiple exoribonucleases participate in the maturation process of the 3′ TAIL^19^. These include *RNase II*, *RNase R, PNPase* and *RNase PH* ^20^, as well as *YbeY* ^21^; hence, the 3′ TAIL is continuously degraded.

Resolving the terminus of the mature 3′ TAIL in *B. subtilis* is the first objective of our study. To this end, we employ high-throughput RNA sequencing (RNA-seq) data. Recent advances in RNA-Seq technologies^22–24^ offer a novel way to identify the 3′ TAIL in the cell by mapping millions of short RNA reads onto the annotated sequence. However, one issue with using RNA-Seq data to analyze the 3′ TAIL is that rRNAs are often removed in the experiments with the use of kits such as RiboMinus from Invitrogen or Ribo-Zero from Epicenter. To circumvent this challenge, we employ publically available datasets for *E. coli* and *B. subtilis* that have not undergone ribo-depletion. We predict that our findings will corroborate the mature 3′ TAIL previously reported^13^. To ensure the fidelity of our method, we analyze *E. coli* data from the same experiment with the expectation of recovering the widely accepted 5′-GAUCACCUCCUUA-3′ reported before^6^.

Determining the non-volatile 3′ end of mature 16S rRNA is crucial to establish 1) correct and meaningful D_toStart_ positioning of the SD/aSD interaction and 2) which nucleotides should be considered when determining the complement SD sequences. Achieving these goals will lead to our second objective: to assess the effects of SD/aSD binding affinity on initiation efficiency while controlling for the optimal D_toStart_ range.

### Determining the optimal SD/aSD interaction that maximizes initiation efficiency

It was generally believed that high SD/aSD binding affinity facilitated translation initiation^25–28^; accordingly, the core aSD motif (CCUCC) was characterized based on its high binding affinity (most negative change in Gibbs free energy [ΔG]). Furthermore, CCUCC is conserved in 277 prokaryotic species using multiple sequence alignment in MAFFT^29^. In practice, putative SD sequences are determined based on their complementarity with an extended sequence at the 3′ TAIL^8–11,30,31^: the inclusion of the core motif CCUCC is canonical, but what constitutes the full extent of the core aSD sequence remains unclear^9^.

The set of identified SD sequences varies depending on the choice of the aSD sequence. A poor set of SD sequences will not provide much insight on initiation efficiency. For example, a recent study^1^ uses 5′-CACCUCC-3′ as the *E. coli* aSD sequence to find putative SD sequences, but observes no correlation between SD binding affinity and translation efficiency. This finding leads to the surprising conclusion that SD/aSD pairing potential has little predictive power over gene expression^30^. A similar study^9^ uses extended aSD sequences (e.g. 5′-ACCUCCUUA-3′ in *E. coli*), and found that intermediate levels of SD/aSD binding maximize translation efficiency, not high binding affinities. This discovery corroborates previous reports^8,32^ showing that SD sequences with intermediate levels of aSD (5′-ACCUCCUU-3′) binding occur most frequently in *E. coli* genes^8^ and that six SD/aSD base pairs lead to more efficient translation and growth than shorter or longer SD/aSD pairs^32^. Taken together, these studies suggest that intermediate levels of SD/aSD binding facilitate the recruitment of the ribosome to the mRNA, but high SD/aSD binding inhibits the transition from initiation to elongation leading to ribosome stalling.

It remains controversial as to what constitutes the core aSD, i.e., the aSD embedded in 3′TAIL that is most frequently involved in functional SD/aSD interactions. We operationally define the core aSD as the sequence motif within 3′TAIL most frequently involved in SD/aSD interactions within optimal D_toStart_ ranges. Although previous studies suggested CCUCC as the core aSD^25–28^, the corroborative reasoning that CCUCC is conserved among bacterial species is a weak one, as 5′-GAUCACCUCCU-3′ is highly conserved among 249 bacterial species^29^, not just CCUCC.

## Results and Discussion

### Elucidating the mature 16S rRNA 3′ tail using RNA-Seq data

**Figure 2:**
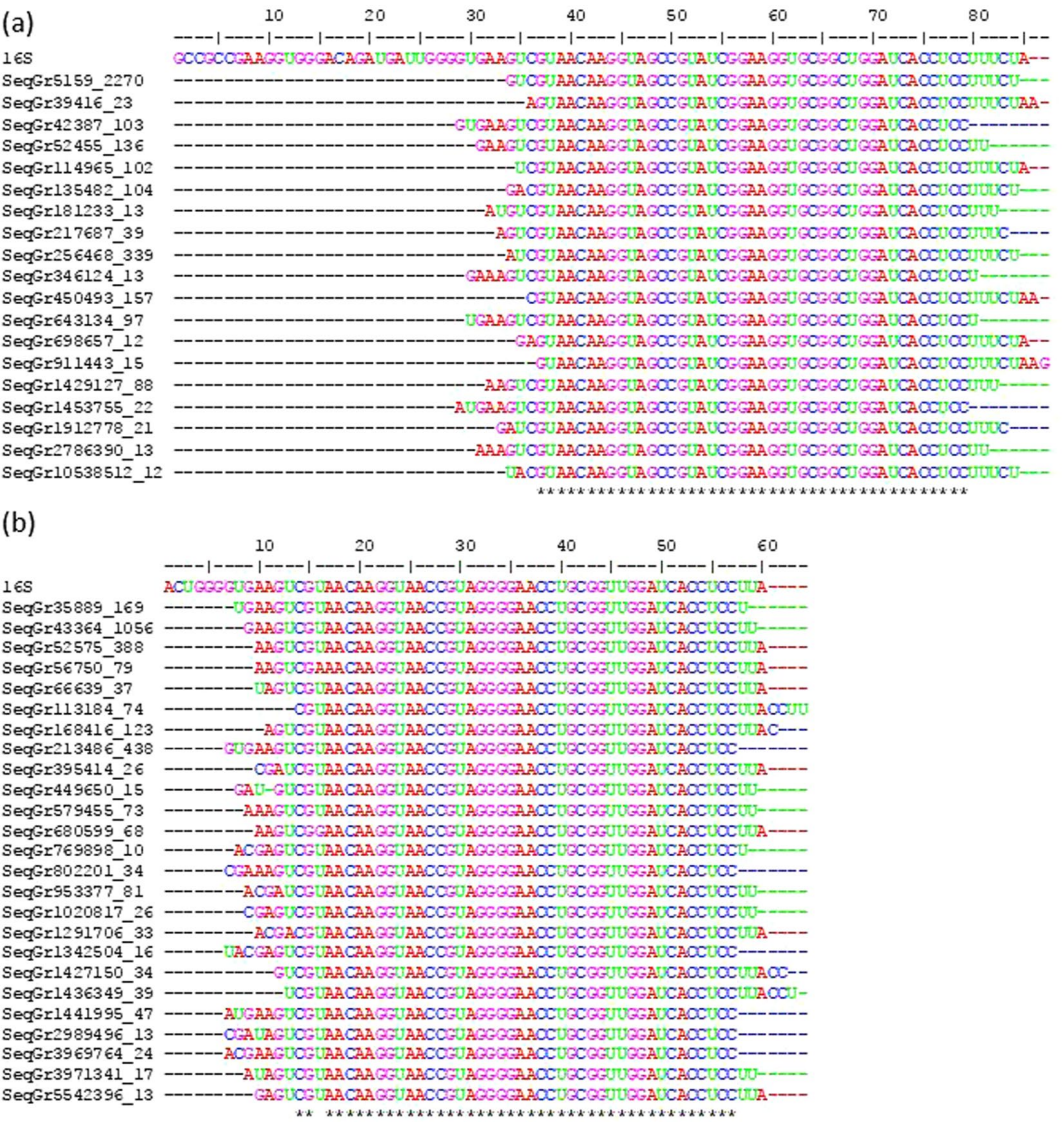
Multiple sequence alignment of reads in FASTA+ format (with sequence ID in the form of ‘ID_##’ where’##’ represents the number of reads that are identical to the represented fragment) matching the 3′ TAIL in (**a**) *B. subtilis* and (**b**) *E. coli*. The top sequence in each panel corresponds to the annotated 3′ TAIL rDNA reference used in BLAST searches from (**a**) NC_000964 and (**b**) NC_000913. Hits were only included in the alignment if they extended to or beyond the 3′ CCUCC motif without base calling errors, and had at least 10 identical matches (accounting for 97.5% of reads in *B. subtilis* and 94% of reads in *E. coli* that fit our criteria).

We identify the 3′ TAIL in *E. coli* and *B. subtilis* using RNA-Seq data. To this end, we BLASTed *B. subtilis* single reads from RNA-Seq run SRR1232437 against 85 nt at the 3′ terminus of the annotated *B. subtilis* 16S rDNA sequence (Fig. 2a, entry labelled 16S, NC_000964). This procedure was also repeated for *E. coli* single reads (SRR1232430) with 60 nt at the 3′ terminus of annotated *E. coli* 16S rDNA sequence (Fig. 2b, entry labelled 16S, NC_000913). We then eliminated BLAST hits that did not extend to encompass the conserved core CCUCC motif of the 3′ TAIL. From the remaining hits, we generated a distribution that indicates the prevalence of 3′ termini (Fig. 2) in both species.

**Figure 3:**
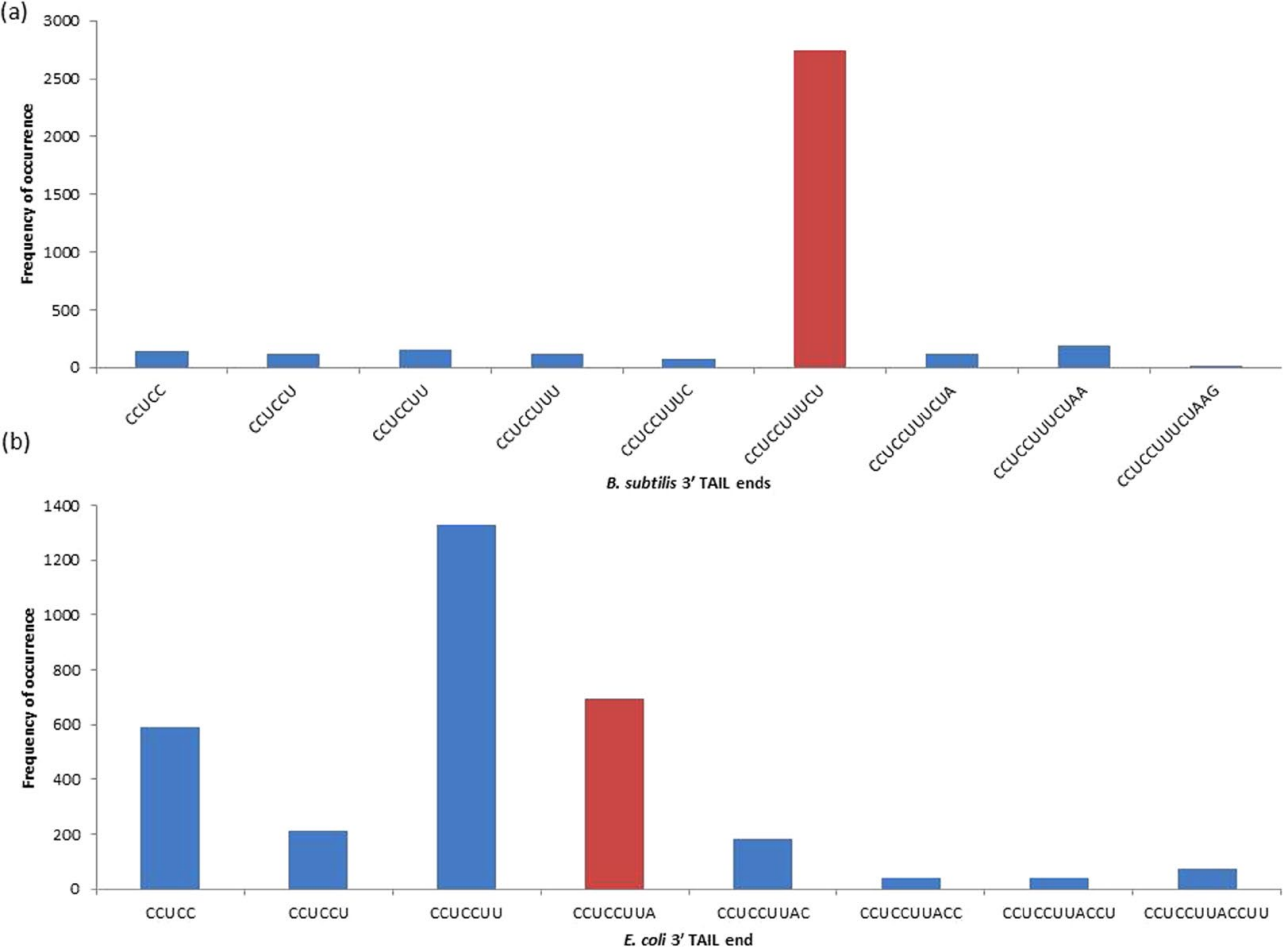
The distribution of hits corresponding to specific 16S rRNA 3′ ends in (**a**) *B. subtilis* and (**b**) *E. coli*. The frequencies of terminal nucleotides for each 3′ TAIL BLAST hit extending to or beyond CCUCC are depicted. Red bars represent the frequencies associated with the first reported 3′ ends in each species.

We expect to recover the universally accepted 3′ terminus reported for *E. coli* ^6^ and, at minimum, the 5′-CCUCCUUUCU-3′ end reported for *B. subtilis* ^13^. In keeping with expectations, the data shows dominant usage of the originally reported 5′-CCUCCUUUCU-3′ end in *B. subtilis* (Fig. 3a), and provides no basis for the inclusion of downstream nucleotides such as A^18^ (NC_000964) or AA (NZ_CP010052) in the mature 3′ TAIL. In contrast, our data suggests characterization of the mature 3′ TAIL in *E. coli* may be less straightforward than previously reported^6^. Figure 3b presents three major 3′ TAIL termini, the longest of which is the widely accepted 5′-CCUCCUUA-3′. Unexpectedly, we also observe high frequencies of reads ending with CCUCC and 5′-CCUCCUU-3′, which suggests that there may be up to three distinct termini for the mature 3′ TAIL in *E. coli*. Importantly, we do recover the expected 3′ end, which indicates that our method works as intended. These observations show that RNA-Seq data is reasonably accurate and can be used to define rRNA termini in the absence of ribo-depletion. Moreover, we propose that the methodology which we apply herein to map the 3′ termini of 16S rRNAs can be extended not only to other species, but also to mapping the termini of other RNA molecules. The RNA-Seq data can be potentially used to characterize transcription start and termination sites as well, paving the way for accurate determination of operons.

It is worth mentioning that the quality of rRNA identification may vary depending on the RNA-Seq protocol used. For instance, when ribo-depletion is employed, although reads mapping to rRNAs may be recovered, the sequence quality is generally poor (Supplementary Fig. S1). Other factors affecting sequence quality include the average read length sequenced in the experiment (sequence quality tends to depreciate towards the end of longer reads), and whether single or paired-end reads are assessed.

Our characterization of the discrete terminus of the mature 3′ TAIL in *B. subtilis* emphasizes that the common practice of approximating the 16S rRNA terminus based on sequence similarity^29,33^ is inadequate. The underlying issue surrounding these instances of poor annotation is the ease with which they are propagated in automated annotation^34,35^. Using such annotations may potentially skew conclusions in studies on translation initiation. For example, investigations considering the *B. subtilis* 3′ TAIL 5′-GAUCACCUCCUUUCUA-3′^1,8–10^ will inherently include a subset of SD/aSD interactions that may detract from the clarity of existing patterns because there can be no translation-mediated selection affecting nucleotides that are absent at the RNA level (the 3′ A). This motivates us to reanalyze optimal SD and aSD sequences in *E. coli* and *B. subtilis* using 3′ TAILs determined by the RNA-Seq data herein.

### The effect of SD/aSD pairing location on initiation efficiency

**Figure 4:**
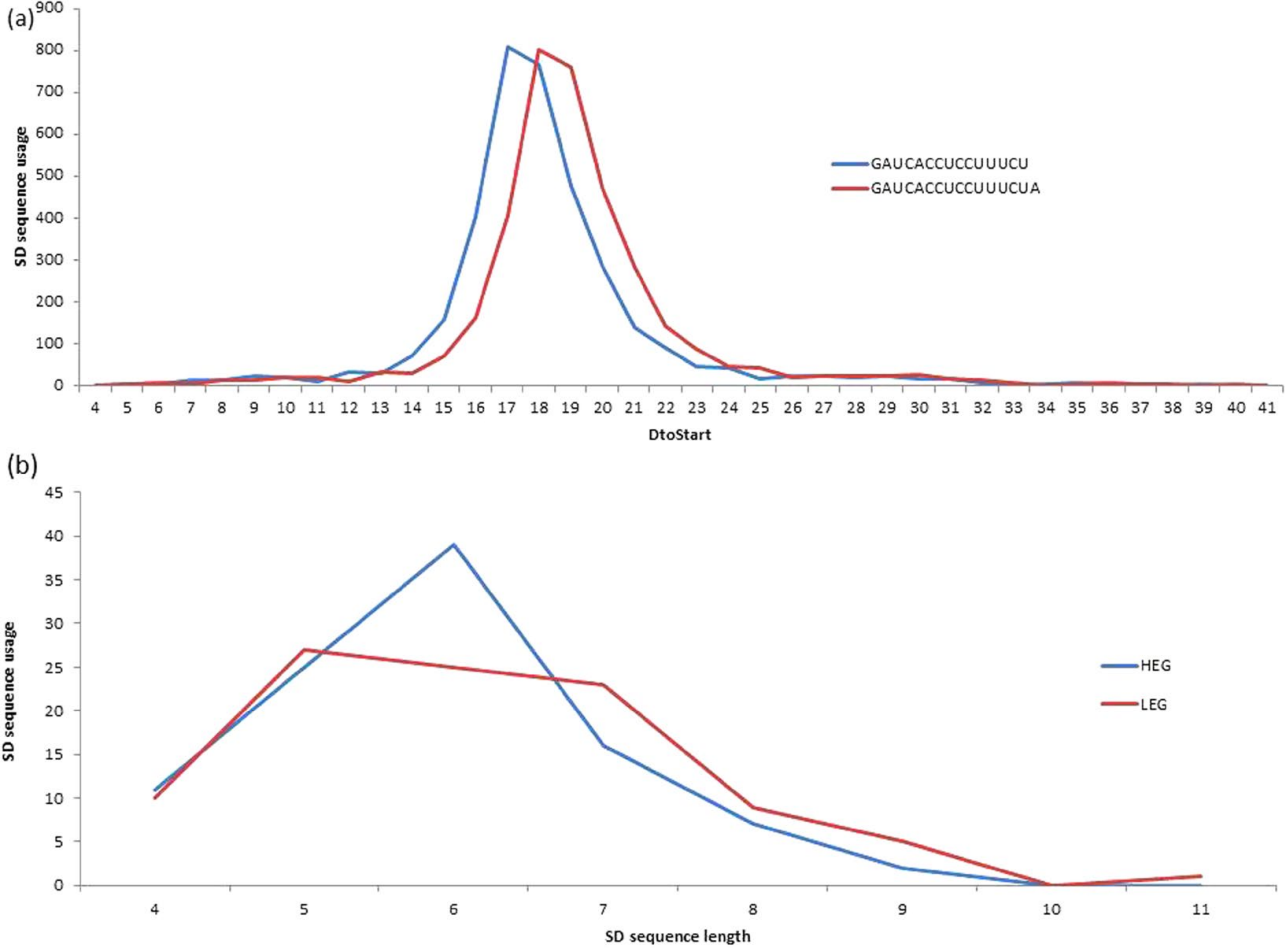
(**a**) D_toStart_ is constrained to a narrow range in all *B. subtilis* putative SD sequences, but the optimal range varies depending on the terminus of the 3′ TAIL. (**b**) Difference in motif length preference of SD sequences with D_toStart_ = 17 in HEGs and LEGs.

The mature 3′ TAIL in *B. subtilis* identified here (5′-GAUCACCUCCUUUCU-3′) requires that the optimal range for D_toStart_ positions, described in a previous study as 15–25 using 5′-GAUCACCUCCUUUCUA-3′^10^, to be redefined. In order to accomplish this, we determined all putative SD sequences between the lengths of 4 and 12 nt (see Materials and Methods for more detail) that complement the mature 3′ TAIL 5′-GAUCACCUCCUUUCU-3′ determined herein. We redefined the optimal range of D_toStart_ distances as 15 to 21 nt in *B. subtilis* based on the optimal range shown in Fig. 4a. As for *E. coli*, the previously reported D_toStart_ range of 10 to 21 nt^10^ was preserved because the same mature 3′ TAIL (5′-GAUCACCUCCUUA-3′) was used.

To clearly highlight the effect of binding affinity on initiation efficiency and show that positioning alone is insufficient to determine optimal SD/aSD pairings, we examine *B. subtilis* putative SD sequences occurring at the most frequently observed distance (Fig. 4a; D_toStart_ = 17). We show a high preference for the usage of six nt motifs in highly expressed genes (HEGs), but not in lowly expressed genes (LEGs) (Fig. 4b). The SD/aSD pairing length is directly associated with binding affinity (longer sequences have higher binding affinity than short sequences), but this association alone is inadequate to capture the heterogeneity intrinsic to a given pair length. For instance, 5′-CCUUU-3′ and CCUCC are both five nt SD sequences that are complementary to the aSD in *B. subtilis*; however, the binding affinity in the former is −3.04 kcal/mol while it is −7.05 kcal/mol in the latter (based on RNAcofold^36^). This implies that, despite having the same pairing length, SD/aSD pairings with CCUCC are substantially more stable than those with 5′-CCUUU-3′. It is for this reason that we explicitly consider binding affinity in the next section.

### Determining the core aSD sequence based on SD/aSD pairing preference

**Figure 5:**
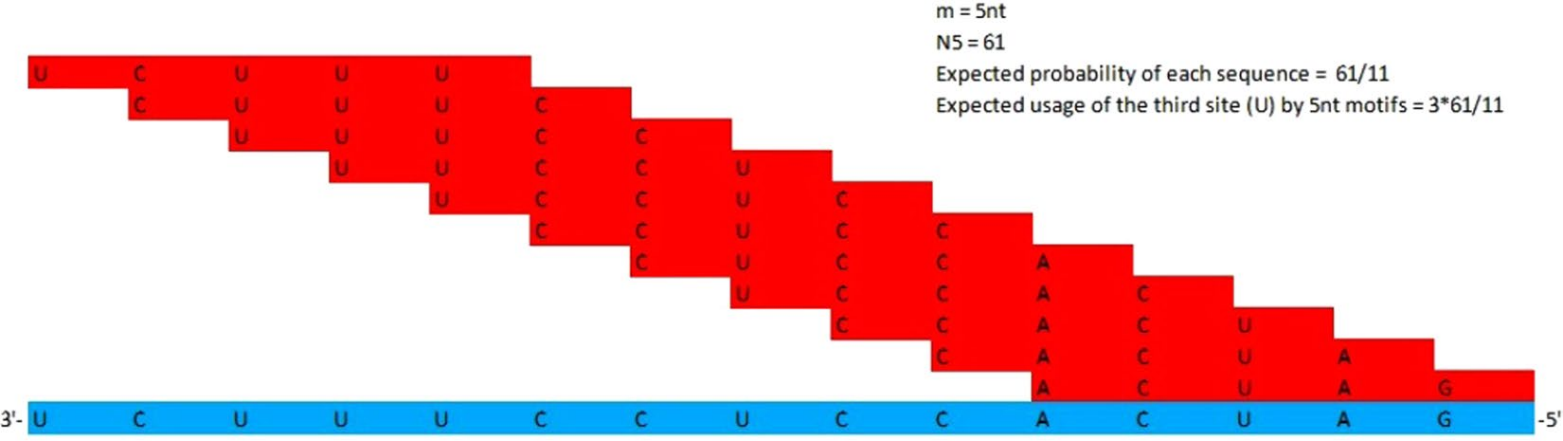
The matching scheme illustrating the expected site specific usage at each aSD site by 5 nt SD sequences (e.g. 61 observed 5 nt SD sequences). Each aSD site (blue) is equally likely to participate in SD/aSD binding with an individual SD sequence (red) assuming there are no site specific selection biases. The aSD site specific expected usage is location-dependent, varying based on displacements of 61 sequences.

**Figure 6:**
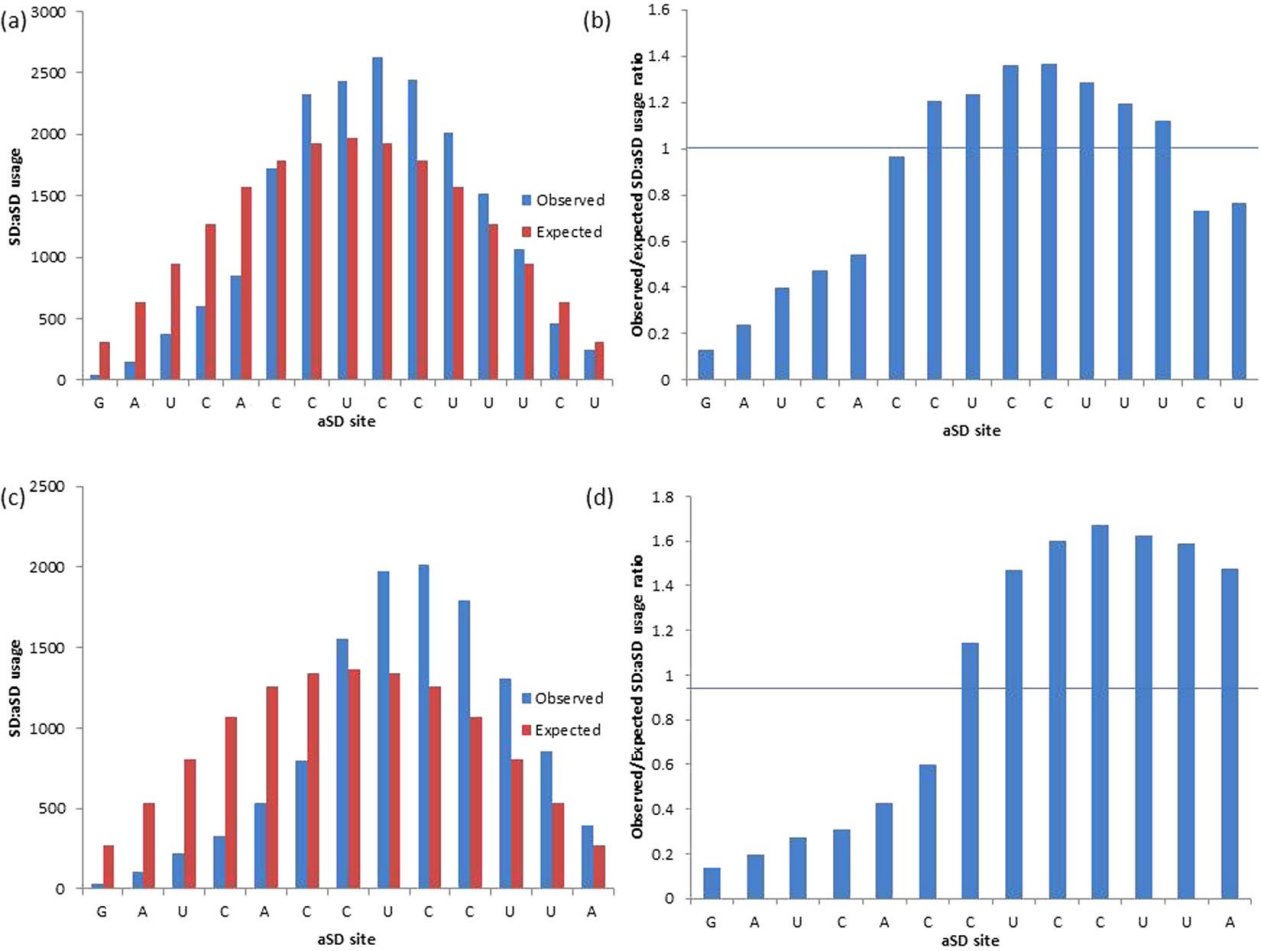
The observed and expected usages and observed/expected usage ratios of aSD sites in (**a** and **b**) *B*. subtilis (5′-GAUCACCUCCUUUCU-3′) and (**c** and **d**) *E. coli* (5′-GAUCACCUCCUUA-3′) by all putative SD sequences. Putative SD sequences (4 to 12 nt) are determined at optimal D_toStart_ locations (15 to 21 in *B. subtilis*, 10 to 21 in *E. coli*).

To determine the extent of the core aSD sequence for both species, we examined the observed and expected usages for each site of the 3′ TAIL in base pairing with all putative SD sequences. To control for the influence of SD/aSD binding location, we only considered putative SD sequences that are located within optimal D_toStart_ ranges discussed previously. For *B. subtilis*, we define bases within the 3′ TAIL 5′-GAUCACCUCCUUUCU-3′ as aSD sites. The expected aSD site usage is estimated assuming that a given SD sequence between four and 12 nt has an equal chance to pair with any given segment within the 3′ TAIL (Fig. 5; See Materials and Methods for more detail). Determining the observed and expected aSD site usages is an important step in examining SD sequence preference. Bases toward the middle of the aSD sequence are more predisposed to pairing with SD sequences than those towards the ends, as illustrated in Fig. 5. Since CCUCC constitutes the middle segment of the 3′ TAIL: 5′-GAUCACCUCCUUA-3′^6^ and 5′-GAUCACCUCCUUUCU-3′^13^ in *E. coli* and *B. subtilis*, respectively, it is unsurprising that the expected usage of this motif is the highest, as illustrated in Fig. 6. Consequently, one must contrast between observed and expected usages of aSD sites to determine their preference and avoidance of SD sequences. In this respect, the shortcoming of Osterman *et al*. (2013) is that they did not contrast the observed and expected SD sequence usages when contrasting sequence occurrences by binding affinity.

We characterize an aSD site to be favorably selected if it pairs with putative SD sequences more frequently than expected (Fig. 6a and c), or has an observed/expected usage ratio >1 (Fig. 6b and d). In *B. subtilis*, aSD sites 5′-CUCCUUU-3′ are favorably selected (Fig. 6a and b), and in *E. coli*, aSD sites 5′-CUCCUUA-3′ were found to be favorably selected (Fig. 6c and d). These results suggest that these sequences make up the extent of the core aSD sequence in the two species. We extended these sequences to 5′-CCUCCUUU-3′ in *B. subtilis* and 5′-CCUCCUUA-3′ in *E. coli* in order to examine the necessity of including the core aSD motif CCUCC in core aSD sequences.

**Figure 7:**
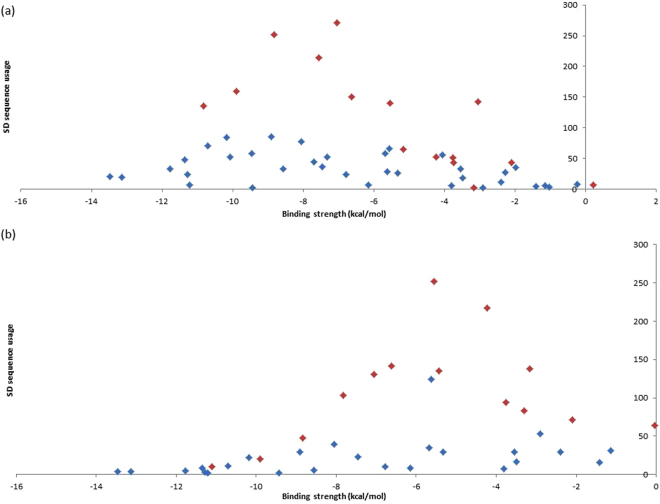
Usages of 4 to 8 nt putative SD sequences and their aSD binding affinity in (**a**) *B. subtilis* and (**b**) *E. coli*. SD sequences with complementarity to the extended core aSD sequences 5′-CCUCCUUU-3′ (*B. subtilis*) and 5′-CCUCCUUA-3′ (*E. coli*) are highlighted red, all other SD sequences are highlighted blue.

**Figure 8:**
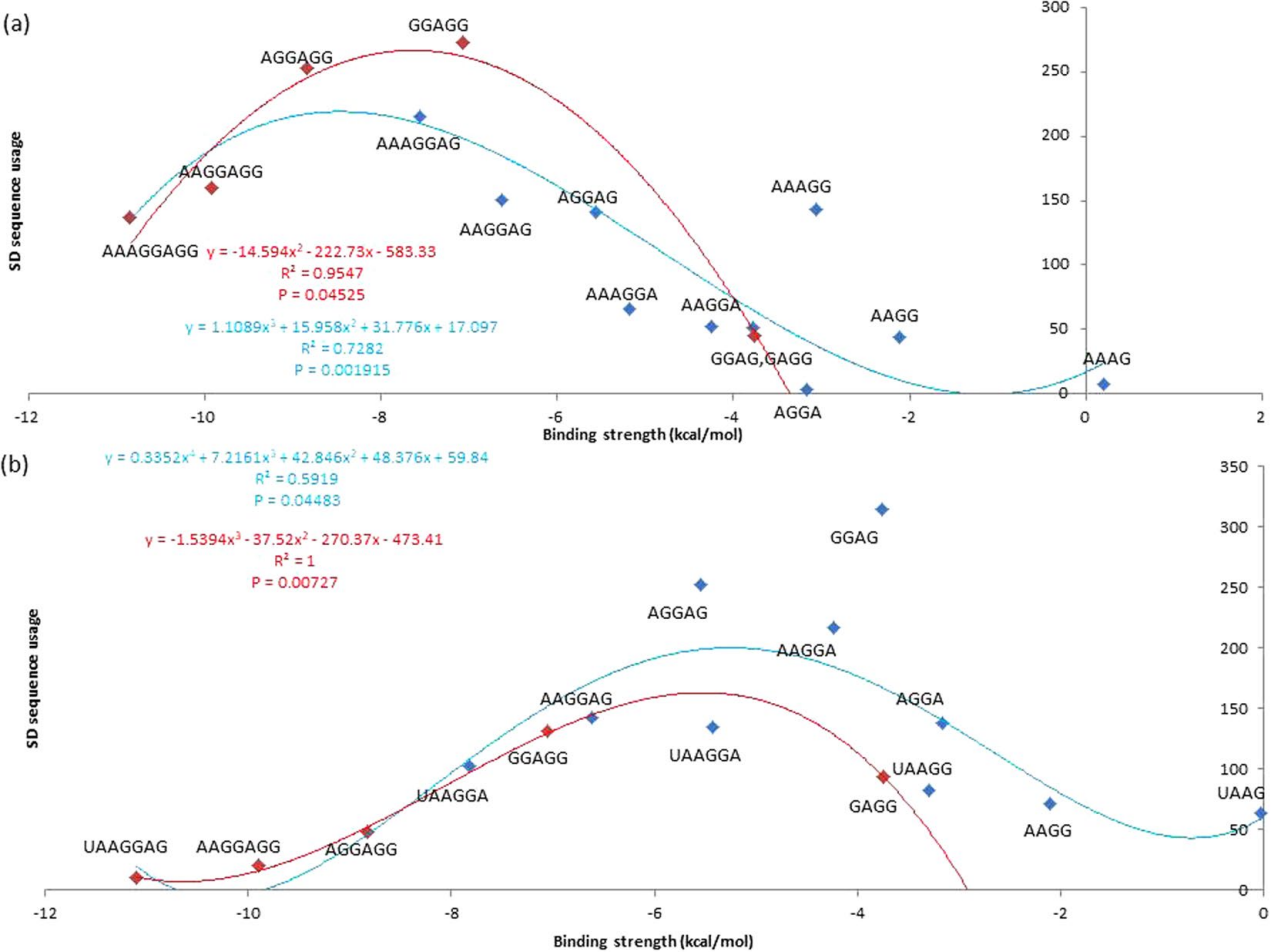
Relationship between the usages of 4 to 8 nt putative SD sequences and their aSD binding affinity in (**a**) *B. subtilis* and (**b**) *E. coli*. All SD sequences have complementarity with the aSD sequences 5′-CCUCCUUU-3′ (*B. subtilis*) and 5′-CCUCCUUA-3′ (*E. coli*). Highlighted in blue are SD sequences that complement only to the un-extended 5′-CUCCUUU-3′ (*B. subtilis*) and 5′-CUCCUUA-3′ (*B. subtilis*). Highlighted in red are SD sequences that were identified after the core aSD sequences were extended to encompass CCUCC.

To investigate whether our core aSD sequences are ideal for translation initiation, we consider their complementary SD sequences. We predicted that 1) putative SD sequences that complement the aforementioned core aSD sequences are favorably selected and constitute the majority of observed SD sequences used by protein-coding genes, and 2) the usage of these SD sequences can be explained by their binding affinities. We found that the most abundant SD sequences used by protein-coding genes are among the four to eight nt putative SD sequences that complement 5′-CCUCCUUU-3′ in *B. subtilis* (Figs. 7a), and 5′-CCUCCUUA-3′ in *E. coli* (Fig. 7b). Furthermore, usages of these SD sequences that complement our core aSD sequences can be explained by their binding affinities (ΔG for heterodimer binding). Specifically, highly used SD sequences have relatively intermediate levels of binding affinities in *B. subtilis* (Fig. 8a: approximately −9 kcal/mol to −7 kcal/mol, P = 0.001915, R^2^ = 0.7282) and in *E. coli* (Fig. 8b: approximately −6 kcal/mol to −4 kcal/mol, P = 0.04483, R^2^ = 0.5919). However, usages of other SD sequences are minimal and cannot be explained by binding affinity. Thus, not all SD sequences with intermediate levels of aSD binding affinities maximize translation efficiency, only the ones that complement the core aSD sequence.

The inclusion of CCUCC in the core aSD sequence depends on the species specific preferred SD/aSD binding affinity; it is not necessarily encompassed by the core aSD sequence of all species. For example, *E. coli* has a lower preferred SD/aSD binding affinity relative to *B. subtilis*, hence SD sequences that complement CCUCC (−7.05 kcal/mol) are less selected for in the former than the latter due to the high binding affinity of the motif (Fig. 8b). Based on these observations, we suggest that the core aSD sequence is extended to 5′-CCUCCUUU-3′ in *B. subtilis*. It should also be noted that the observed/expected ratio at the 5′ C is very close to one (the base is not avoided by SD sequences; Fig. 6B).

**Figure 9:**
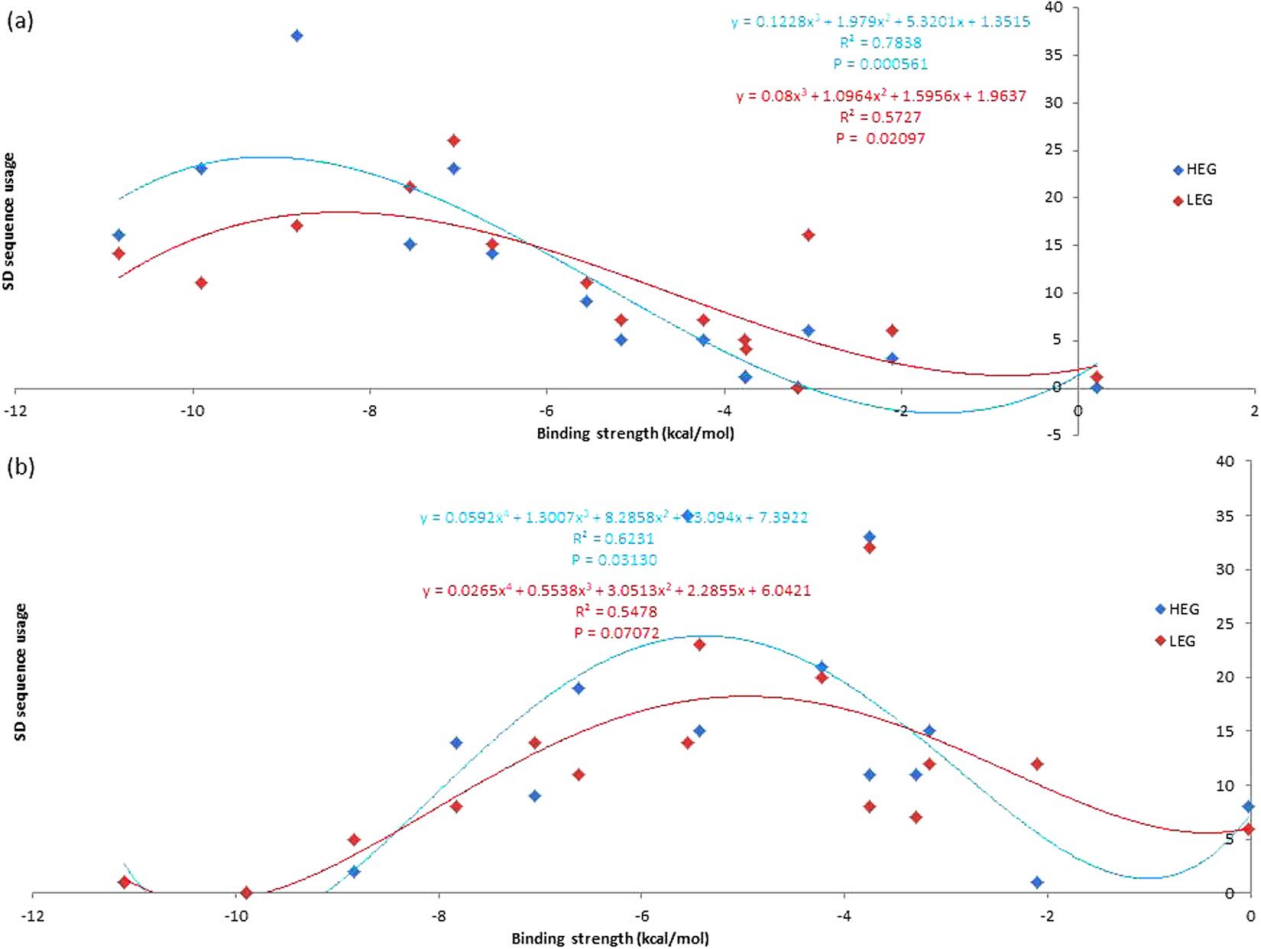
The association between SD sequence usage and binding affinity is more pronounced in HEGs than LEGs in (**a**) *B. subtilis* and (**b**) *E. coli*. All 4 to 8 nt SD sequences are complementary to aSD sequences CCUCCUUU-3′ (*B. subtilis*) and 5′-CCUCCUUA-3′ (*E. coli*).

We expect the association between SD sequence usage and binding affinity to be more pronounced in HEGs than LEGs. Indeed, SD sequences with relatively intermediate levels of binding affinity are more preferred in HEGs than LEGs in both *B. subtilis* and *E. coli* (Fig. 9). This contrast further emphasizes the importance of SD binding affinity in translation efficiency because HEGs are under greater selective pressure to evolve towards high translation efficiency than LEGs. This finding complements the claim made by Hockenberry *et al*.^9^ that translation efficiency is maximized at intermediate levels of SD/aSD binding affinity, and extends their conclusion to suggest that intermediate SD binding affinities are preferred in both HEGs and LEGs.

We suggest that optimal SD sequences are 5′-AGGAGG-3′ and 5′-AAAGGAG-3′ in *B. subtilis*, and 5′-AGGAG-3′ and 5′-GGAG-3′ in *E. coli* (Fig. 9), based on their 1) high usages, especially in HEGs, 2) intermediate binding affinity to core aSD sequences (5′-CCUCCUUU-3′ in *B. subtilis*, and 5′-CUCCUUA-3′ in *E. coli*), and 3) occurrences at optimal D_toStart_ locations. Elucidating the full extent of the core aSD sequence is important to identify the complete set of optimal SD/aSD pairs. For example, one would not be able to detect the highly preferred SD sequences 5′-AGGAG-3′, 5′-AAGGA-3′ and 5′-GGAG-3′ in *E. coli* using the aSD sequence 5′-CACCUCC-3′. This explains why no correlation was observed between SD binding affinity and translation efficiency in a previous study^1^. On the other hand, one will overestimate the amount of different SD sequences by extending past the core aSD sequence at either end. The usages of such SD sequences are not preferred and cannot be explained by binding affinity; they are likely poor motifs for translation initiation. Lastly, we acknowledge that there is considerable flexibility in the SD sequence (perfect complementarity is not necessary between SD and aSD bases). We speculate that this is due to the fact that intermediate levels of binding affinity are preferable.

## Materials and Methods

### Processing the genome and RNA-Seq data

The annotated genomes of *B. subtilis 168* (accession number: NC_000964) and *E. coli* K12 (NC_000913) in GenBank formats were retrieved from the National Center for Biotechnology Information (NCBI) database (http://www.ncbi.nlm.nih.gov). Two FASTQ files in BioProject PRJNA244362 (*B. subtilis 168* wild type, experiment SRX515181, sequencing length ~ 51 nt) and (*E. coli K12* wild type experiment SRX515174, sequencing length ~ 51 nt) were downloaded from NCBI and converted into FASTA files using seqtk (https://github.com/lh3/seqtk), then subsequently into FASTA+ format using ARSDA^37^ (http://dambe.bio.uottawa.ca/Include/software.aspx). The site specific qualities of RNA-Seq reads were visualized in ARSDA via the ‘Get.FASTQ Info′ from the FASTQ files.

### Aligning RNA-Seq reads to annotated rRNA sequences

The FASTA+ files were converted into BLAST databases using the “Create BLAST DB” function in ARSDA. Annotated segments of the 3′ 16S rDNA were used as the query sequences (the final 85 nt of 16S rRNA in *B. subtilis* accession NC_000964 and the final 60 nt of the 16S rRNA in accession NC_000913) for BLAST alignments against the generated BLAST databases (both using specified e-value cutoffs of 10^−17^ and word length = 20). The resulting hits were retrieved from the FASTA+ files using DAMBE and aligned by multiple sequence alignment (using the Clustal Omega algorithm implemented in DAMBE, default parameters) against the corresponding 16S segment for each organism. Reads were retained if they extended to at least the final C in the canonical CCUCC motif and had no errors in base calling towards the 3′ ends. All reads that match these criteria were used in generating the distributions shown in Fig. 2.

### Classifying genes according to gene expression

We used protein abundance (ppm) data as proxies of gene expression. The integrated datasets were downloaded from PaxDB^38^ for *E. coli* and *B. subtilis*. The *B. subtilis* protein IDs (224308-paxdb_uniprot.txt) were mapped to Gene IDs in NC_000964 using UniProt Retrieve/ID mapping http://www.uniprot.org/uploadlists/. The *E. coli* protein IDs were in the same format as the Gene IDs in NC_000913. The genes were ranked by protein abundance values, and the top and bottom 10% of the genes were classified as HEGs and LEGs, respectively. Only genes with non-zero protein abundance values were selected in this study.

### Determining putative SD sequences based on pairing potential, location, and binding affinities

The 3′ TAILs 5′-GAUCACCUCCUUUCU-3′ (*B. subtilis*) and 5′-GAUCACCUCCUUA-3′ (*E. coli*) were used in identifying putative SD sequences using DAMBE^39^, following the methods used in two previous studies^10,11^: 30 nt upstream of start codon of all CDSs were extracted and matched against the annotated 3′ TAIL with ‘Analyzing 5′UTR’ in DAMBE, with minimum SD length = 4 nt and maximum SD length = 12 nt. The SD/aSD binding affinities (ΔG for heterodimer binding) were calculated using RNAcofold with default settings^36^.

Only SD sequences occurring at optimal distances relative to the start codon were analyzed in this study. The optimal distances for SD sequences were determined to be 10 to 21 D_toStart_ bases in *E. coli* ^10^ and 15 to 21 D_toStart_ bases in *B. subtilis*. D_toStart_ denotes the distance between the 16S rRNA 3′ end and the start codon during SD/aSD binding.

### Calculating the SD/aSD observed and expected site specific usage

The observed usage of each *B. subtilis* SD site represents the total number of times the base is observed in all putative *B. subtilis* SD sequences of protein-coding genes and of highly and lowly expressed subsets of genes. The expected usage of each SD site represents the total number of times the base is expected to occur in putative SD sequences, assuming each SD site is equally likely to be used by all SD sequences of lengths 4 nt to 12 nt (no selection bias). Thus, the expected number of SD/aSD binding at the first aSD site is represented by equation (1), with N_m_ denoting N observed number of SD sequences of length m:

1 $$\sum _{m=4}^{12}\frac{{N}_{m}}{15-m+1}\,$$

While the expected frequency at the sixth aSD site is represented by equation (2):

2 $$4\times \frac{{N}_{4}}{12}+5\times \frac{{N}_{5}}{11}+6\times \frac{{N}_{6}}{10}+6\times \frac{{N}_{7}}{9}+6\times \frac{{N}_{8}}{8}+6\times \frac{{N}_{9}}{7}+6\times \frac{{N}_{10}}{6}+6\times \frac{{N}_{11}}{5}+5\times \frac{{N}_{12}}{4}$$

The same methodology is applied to measure usage of *E*. coli SD sequences. These computations are implemented in DAMBE^39,40^ under the ‘Analyze 5UTR’ function.

### Data availability

All data used in our analyses are publicly available in the file Supplementary Dataset 1. Raw data are extracted from the NCBI GEO DataSets database (https://www.ncbi.nlm.nih.gov/gds). The runs used for *B. subtilis* (SRR1232437) and *E*. *coli* (SRR1232430) are both included under accession GSE56720. The integrated protein abundance data are available at PaxDB (https://pax-db.org/).

## Electronic supplementary material

Supplementary Information

Supplementary Dataset 1

## References

[1] Li GW, Burkhardt D, Gross C, Weissman JS (2014). Quantifying absolute protein synthesis rates reveals principles underlying allocation of cellular resources. Cell.

[2] Kudla G, Murray AW, Tollervey D, Plotkin JB (2009). Coding-Sequence Determinants of Gene Expression in Escherichia coli. Science.

[3] Tuller T, Waldman YY, Kupiec M, Ruppin E (2010). Translation efficiency is determined by both codon bias and folding energy. Proc. Natl. Acad. Sci. USA.

[4] Xia X (2015). A Major Controversy in Codon-Anticodon Adaptation Resolved by a New Codon Usage Index. Genetics.

[5] Walsh G (2005). Therapeutic insulins and their large-scale manufacture. Appl. Microbiol. Biotechnol..

[6] Shine J, Dalgarno L (1974). The 3′-terminal sequence of Escherichia coli 16S ribosomal RNA: complementarity to nonsense triplets and ribosome binding sites. Proc. Natl. Acad. Sci. USA.

[7] Hui A, de Boer HA (1987). Specialized ribosome system: preferential translation of a single mRNA species by a subpopulation of mutated ribosomes in Escherichia coli. Proc. Natl. Acad. Sci. USA.

[8] Osterman IA, Evfratov SA, Sergiev PV, Dontsova OA (2013). Comparison of mRNA features affecting translation initiation and reinitiation. Nucleic Acids Res.

[9] Hockenberry, A. J., Pah, A. R., Jewett, M. C. & Amaral, L. A. Leveraging genome-wide datasets to quantify the functional role of the anti-Shine-Dalgarno sequence in regulating translation efficiency. *Open biology* **7**, 10.1098/rsob.160239 (2017). 10.1098/rsob.160239 PMC5303271 28100663

[10] Abolbaghaei A, Silke JR, Xia X (2017). How Changes in Anti-SD Sequences Would Affect SD Sequences in *Escherichia coli* and Bacillus subtilis. G3 (Bethesda, Md.).

[11] Prabhakaran R, Chithambaram S, Xia X (2015). Escherichia coli and Staphylococcus phages: effect of translation initiation efficiency on differential codon adaptation mediated by virulent and temperate lifestyles. J Gen Virol.

[12] de Smit MH, van Duin J (1994). Translational initiation on structured messengers. Another role for the Shine-Dalgarno interaction. J Mol Biol.

[13] Murray CL, Rabinowitz JC (1982). Nucleotide sequences of transcription and translation initiation regions in Bacillus phage phi 29 early genes. J. Biol. Chem..

[14] Green CJ, Stewart GC, Hollis MA, Vold BS, Bott KF (1985). Nucleotide sequence of the Bacillus subtilis ribosomal RNA operon, rrnB. Gene.

[15] Uchida T (1974). The use of ribonuclease U2 in RNA sequence determination. Some corrections in the catalog of oligomers produced by ribonuclease T1 digestion of Escherichia coli 16S ribosomal RNA. J. Mol. Evol..

[16] Woese CR (1975). Conservation of primary structure in 16S ribosomal RNA. Nature.

[17] Barbe V (2009). From a consortium sequence to a unified sequence: the Bacillus subtilis 168 reference genome a decade later. Microbiology.

[18] Sohmen D (2015). Structure of the Bacillus subtilis 70S ribosome reveals the basis for species-specific stalling. Nature communications.

[19] Deutscher MP (2015). Twenty years of bacterial RNases and RNA processing: how we’ve matured. RNA.

[20] Sulthana S, Deutscher MP (2013). Multiple exoribonucleases catalyze maturation of the 3′ terminus of 16S ribosomal RNA (rRNA). J. Biol. Chem..

[21] Jacob AI, Kohrer C, Davies BW, RajBhandary UL, Walker GC (2013). Conserved bacterial RNase YbeY plays key roles in 70S ribosome quality control and 16S rRNA maturation. Mol Cell.

[22] Wang Z, Gerstein M, Snyder M (2009). RNA-Seq: a revolutionary tool for transcriptomics. Nat Rev Genet.

[23] Mortazavi A, Williams BA, McCue K, Schaeffer L, Wold B (2008). Mapping and quantifying mammalian transcriptomes by RNA-Seq. Nat Methods.

[24] Li S, Dong X, Su Z (2013). Directional RNA-seq reveals highly complex condition-dependent transcriptomes in E. coli K12 through accurate full-length transcripts assembling. BMC Genomics.

[25] Lim K, Furuta Y, Kobayashi I (2012). Large variations in bacterial ribosomal RNA genes. Mol Biol Evol.

[26] Ma J, Campbell A, Karlin S (2002). Correlations between Shine-Dalgarno sequences and gene features such as predicted expression levels and operon structures. J Bacteriol.

[27] Schurr T, Nadir E, Margalit H (1993). Identification and characterization of E.coli ribosomal binding sites by free energy computation. Nucleic Acids Res.

[28] Starmer J, Stomp A, Vouk M, Bitzer D (2006). Predicting Shine-Dalgarno Sequence Locations Exposes Genome Annotation Errors. PLoS Comput Biol.

[29] Nakagawa S, Niimura Y, Miura K, Gojobori T (2010). Dynamic evolution of translation initiation mechanisms in prokaryotes. Proc. Natl. Acad. Sci. USA.

[30] Li GW (2015). How do bacteria tune translation efficiency?. Curr Opin Microbiol.

[31] Li GW, Oh E, Weissman JS (2012). The anti-Shine-Dalgarno sequence drives translational pausing and codon choice in bacteria. Nature.

[32] Vimberg V, Tats A, Remm M, Tenson T (2007). Translation initiation region sequence preferences in *Escherichia coli*. BMC Mol Biol.

[33] Lin YH, Chang BC, Chiang PW, Tang SL (2008). Questionable 16S ribosomal RNA gene annotations are frequent in completed microbial genomes. Gene.

[34] Jones CE, Brown AL, Baumann U (2007). Estimating the annotation error rate of curated GO database sequence annotations. BMC Bioinformatics.

[35] Lagesen K (2007). RNAmmer: consistent and rapid annotation of ribosomal RNA genes. Nucleic Acids Res.

[36] Lorenz R (2011). ViennaRNA Package 2.0. Algorithms Mol Biol.

[37] Xia X (2017). ARSDA: A New Approach for Storing, Transmitting and Analyzing Transcriptomic Data. G3: Genes|Genomes|Genetics.

[38] Wang M, Herrmann CJ, Simonovic M, Szklarczyk D, von Mering C (2015). Version 4.0 of PaxDb: Protein abundance data, integrated across model organisms, tissues, and cell-lines. Proteomics.

[39] Xia X (2017). DAMBE6: New Tools for Microbial Genomics, Phylogenetics, and Molecular Evolution. J Hered.

[40] Xia X (2013). DAMBE5: A comprehensive software package for data analysis in molecular biology and evolution. Mol Biol Evol.

