# Supplementary material for: Elucidating the 16S rRNA 3′ boundaries and defining optimal SD/aSD pairing in Escherichia coli and Bacillus subtilis using RNA-Seq data
Source: Sci Rep. 2017 Dec 15;7:17639. doi: 10.1038/s41598-017-17918-6 (PMC5732282; doi:10.1038/s41598-017-17918-6)
Supplement: Supplementary file 1 — Supplementary Information [file 41598_2017_17918_MOESM1_ESM.pdf]

Elucidating the 16S rRNA 3' boundaries and defining optimal SD/aSD pairing in *Escherichia coli* and *Bacillus subtilis* using RNA-Seq data

Yulong Wei[1†], Jordan R. Silke[1†], and Xuhua Xia[12*]

1. Department of Biology, University of Ottawa, 30 Marie Curie, P.O. Box 450, Station A, Ottawa, Ontario, Canada, K1N 6N5. Tel: (613) 562-5800 ext. 6886, Fax: (613) 562-5486.

2. Ottawa Institute of Systems Biology, Ottawa, Ontario, Canada K1H 8M5.

† Equal contribution.

* Corresponding author: E-mail: xxia@uottawa.ca

Supplementary Information

```
                        10        20        30        40        50        60        70        80
               ----|----|----|----|----|----|----|----|----|----|----|----|----|----|----|----|-
SeqGr119948_1  -----UUUUGAUUCAUGACUGGGGUGAAGUCGUAACAAGGUAACCGUAGGGGAACCUGCGGUUGGAUCACCUCCUCUCCC-
SeqGr1450749_4 ----UUUGUGAUUCAUGACUGGGGUGAAGUCGUAACAAGGUAACCGUAGGGGAACCUGCGGUUGGAUCACCUCCUCUCC--
SeqGr1496175_3 -----UUGUGAUUCAUGACUGGGGUGAAGUCGUAACAAGGUAACCGUAGGGGAACCUGCGGUUGGAUCACCUCCUCUCCC-
SeqGr3193684_1 --AAGGUGGGACAGAUGAUUGGGGUGAAGUCGAAACAAGGUAGCCGUAUCGGAAGGUGCGGCUGGAUCACCUCCUUU----
SeqGr3837735_1 --UUGGUGGGACAGAUGAUUGGGGUGAAGUCGAAACAAGGUAGCCGUAUCGGAAGGUGCGGCUGGAUCACCUCCUUU----
SeqGr3961119_1 ---CUUUGUGAUUCAUGAAUGGGGUGAAGUCGUAACAAGGUAACCGUAGGGGAACCUGCGGUUGGAUCACCUCCUCUC---
SeqGr452173_1  ---UUUUGUGAUUCAUGACUGGGGUGAAGUCGUAACAAGGUAACCGUAGGGGAACCUGCGGUUGGAUCACCUCCUCUC---
SeqGr4611819_1 CCACUUUGUGAUUCAUGACUGGGGUGAAGUCGUAACAAGGUAACCGUAGGGGAACCUGCGGUUGGAUCACCUCCU------
SeqGr46365_22  ---CUUUGUGAUUCAUGACUGGGGUGAAGUCGUAACAAGGUAACCGUAGGGGAACCUGCGGUUGGAUCACCUCCUCUC---
SeqGr509358_11 --ACUUUGUGAUUCAUGACUGGGGUGAAGUCGUAACAAGGUAACCGUAGGGGAACCUGCGGUUGGAUCACCUCCUCU----
SeqGr69143_4   -CACUUUGUGAUUCAUGACUGGGGUGAAGUCGUAACAAGGUAACCGUAGGGGAACCUGCGGUUGGAUCACCUCCUC-----
SeqGr134077_56 ---CUUUGUGAUUCAUGACUGGGGUGAAGUCGUAACAAGGUAACCGUAGGGGAACCUGCGGUUGGAUCACCUCCUCUC---
SeqGr144116_27 --ACUUUGUGAUUCAUGACUGGGGUGAAGUCGUAACAAGGUAACCGUAGGGGAACCUGCGGUUGGAUCACCUCCUCU----
SeqGr1815518_1 -CACUUUGUGAAUCAUGACUGGGGUGAAGUCGUAACAAGGUAACCGUAGGGGAACCUGCGGUUGGAUCACCUCCUC-----
SeqGr1970881_3 CCACUUUGUGAUUCAUGACUGGGGUGAAGUCGUAACAAGGUAACCGUAGGGGAACCUGCGGUUGGAUCACCUCCU------
SeqGr2342583_1 --ACAUUGUGUUUCGUGACUGGGGUGAAGUCGUAACAAGGUAACCGUAGGGGAACCUGCGGUUGGAUCACCUCCUCU----
SeqGr2989770_1 ------UGUGAUUCAUGACUGGGGUGAAGUCGUAACAAGGUAACCGUAGGGGAACCUGCGGUUGGAUCACCUCCUCUCCCC
SeqGr3159133_1 ---UUUUGUGAUUCAUGACUGGGGUGAAGUCGUAACAAGGUAACCGUAGGGGAACCUGCGGGUGGAUCACCUCCUCUC---
SeqGr3279744_1 ---CUUUGUGAUUCAUGACUGGGGUGAAGUCGUAACAAGGUAACCGUAGUGGAACCUGCGGUUGGAUCACCUCCUCUC---
SeqGr3543924_1 --AUGGUGGGACAGAUGAUUGGGGUGAAGUCGAAACAAGGUAGCCGUAUCGGAAGGUGCGGCUGGAGCACCUCCUUU----
SeqGr366483_1  ---CUUUGUGAUUCAUGACUGGGGUGAAGUCGUAACAAGGUAACCGUAGGGGAACCUGCGGGUGGAUCACCUCCUCUC---
SeqGr448293_2  -----UUGUGAUUCAUGACUGGGGUGAAGUCGUAACAAGGUAACCGUAGGGGAACCUGCGGUUGGAUCACCUCCUCUCCC-
SeqGr682676_8  ----UUUGUGAUUCAUGACUGGGGUGAAGUCGUAACAAGGUAACCGUAGGGGAACCUGCGGUUGGAUCACCUCCUCUCC--
SeqGr78824_7   -CACUUUGUGAUUCAUGACUGGGGUGAAGUCGUAACAAGGUAACCGUAGGGGAACCUGCGGUUGGAUCACCUCCUC-----
SeqGr841891_1  --AAGGUGGGACAGAUGAUUGGUGUGAAGUCGAAACAAGGUAGCCGUAUCGGAAGGUGCGGCUGGAUCACCUCCUUU----
SeqGr851203_1  ---CUUAGUGAUUCAUGACUGGGGUGAAGUCGUAACAAGGUAACCGUAGGGGAACCUGCGGUUGGAUCACCUCCUCUC---
SeqGr97624_1   ---UUUUUUUUUCAUGACUGGGGUGAAGUCGUAACAAGGUAACCGUAGGGGAACCUGCGGUUGGAUCACCUCCUCUC---
SeqGr1024682_2 ---CUUUGCGAUUCAUGACUGGGGUGAAGUCGUAACAAGGUAACCGUAGGGGAACCUGCGGUUGGAUCACCUCCUCUC---
SeqGr1054000_2 ---UUUUGUGAUUCAUGACUGGGGUGAAGUCGUAACAAGGUAACCGUAGGGGAACCUGCGGUUGGAUCACCUCCUCUC---
rrnA16S        CGAAGGUGGGACAGAUGAUUGGGGUGAAGUCGUAACAAGGUAGCCGUAUCGGAAGGUGCGGCUGGAUCACCUCCUUUCUA-
```

Figure S1. Sequence alignment between the annotated 16S rRNA (rrnA16S, NC_000964) and 75 nt RNA-Seq reads (SRR3466199). RNA-Seq segment IDs are in FASTA+ format (e.g. SeqGr134077_56 represents the 134077th unique sequence, and it has 56 identical copies).
